# Reading graphic medicine at the National Library of Medicine

**DOI:** 10.5195/jmla.2018.449

**Published:** 2018-07-01

**Authors:** Patricia Tuohy, Judith Eannarino

**Affiliations:** Head, Exhibition Program, US National Library of Medicine, National Institutes of Health, Bethesda, MD; Head, Selection Unit, Collection Development and Acquisitions Section, US National Library of Medicine, National Institutes of Health, Bethesda, MD

## Abstract

The Exhibition Program, part of the History of Medicine Division of the National Library of Medicine, spotlights the collection of the library by creating exhibitions and educational resources that explore the social and cultural history of medicine. Our goal is to stimulate people’s enthusiasm for history and encourage visitors of all ages to learn more about themselves and their communities. We do what we do because we believe that health and well-being are fundamental human rights and are essential to our American way of life. And we believe exhibitions are a logical expression of that commitment.

Oftentimes, exhibitions focus on underrepresented subjects or lesser-known types of literature, which helps to inform the library’s collection development activity. Collection development staff take a keen interest in viewing exhibitions, attending related lectures, and performing bibliographic research on topics that are unlikely to be captured in conventional scientific and professional literature. This heightened awareness leads staff to discover niche publishers, significant authors, and unique titles, thereby enriching the collection for future generations.

Following the decision to embark on an exhibition about graphic medicine, collections staff more closely investigated this class of literature. This column explores how wider social and cultural influences can change the medical literature and inform and enrich the collections policies of an institution.

The Exhibition Program, part of the History of Medicine Division of the National Library of Medicine (NLM), spotlights NLM collections by creating exhibitions and educational resources that explore the social and cultural history of medicine. The program’s goal for showcasing NLM’s collections in exhibitions is to stimulate people’s enthusiasm for history and encourage visitors of all ages to learn more about themselves and their communities. We do what we do because we believe that health and well-being are fundamental human rights and are essential to our American way of life, and we believe exhibitions are a logical expression of that commitment.

In the series of exhibitions that examine the relationship between medicine and the arts, NLM launched a new project: *Graphic Medicine: Ill-Conceived and Well-Drawn!* This exhibition explores graphic medicine, an emerging field of medical literature in which patients and their loved ones, caregivers, and health professionals tell stories about health and illness through the medium of comics. In graphic medicine, artists and authors use a combination of words and images to present powerful and informative narratives and understandable health information to their audiences. For patients, graphic medicine promotes health literacy and often provides a therapeutic artistic experience. For health professionals, the genre serves as a gateway to understanding the patient experience and fosters effective treatment strategies.

Writing in 2012 for *Medical Humanities,* physician, educator, artist, and graphic medicine pioneer Ian C. M. Williams published a background and overview of the field. In his article “Graphic Medicine: Comics as Medical Narrative,” Williams asserted:

The comics medium, due to its unique and specific properties, is ideally suited to portraying the subjective experiences of the author with regard to illness and suffering and is furthermore ideally suited to the education of both the public and professionals and that published graphic memoirs of suffering may be of help to the similarly afflicted, or careers and family of the ill. [1]

## COLLECTION DEVELOPMENT

NLM exhibitions often focus on underrepresented subjects or lesser-known types of literature, which helps to inform NLM’s collection development activity. Collection development staff take a keen interest in viewing exhibitions, attending related History of Medicine Division lectures and special display tours, and performing bibliographic research on topics that are unlikely to be captured in conventional scientific and professional literature. This heightened awareness leads staff to discover niche publishers, significant authors, and unique titles, thereby enriching the collection for future generations.

## FIRST STEPS

NLM collection development staff initially encountered comics as literature in the 1990s—somewhat by accident. The first modern graphic works that staff reviewed were manga-style comics produced for Japanese health professionals and medical students. Collection development staff, which included an East Asian specialist, received a few such publications. After a lively discussion, staff decided to collect five examples of these Japanese-language monographs because of their unique cultural and historical value.

Although there were a few contemporary, break-out graphic novels developed for Western audiences (Will Eisner’s *A Contract with God and Other Tenement Stories,* 1978; Art Spiegelman’s *Maus,* 1986; Alan Moore and Dave Gibbons’s *Watchmen,* 1987), it was not foreseeable that the publication of graphic nonfiction would expand to the medical arena and find acceptance by an American audience.

**Figure 1:**
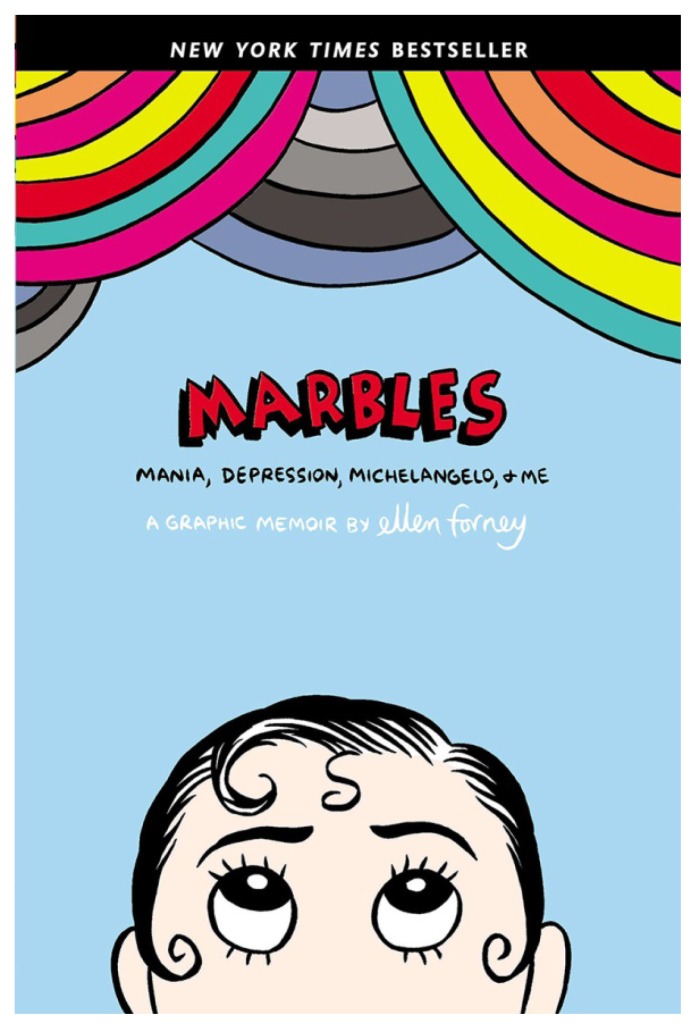
Cover of Marbles: Mania, Depression, Michelangelo, and Me by Ellen Forney, 2012 Used with permission from the author.

Fast forward to 2014: the Exhibition Program staff began researching the possibility of developing a project that would become *Graphic Medicine: Ill-Conceived and Well-Drawn!* For this exhibition, we wanted to work with a guest curator who would be able to communicate the power of graphic medicine to diverse audiences in an exhibition format. Who better to collaborate with than an award-winning graphic medicine author and artist? We invited Ellen Forney—who published the highly regarded graphic medicine work about her struggles with bipolar disorder and creativity, *Marbles: Mania, Depression, Michelangelo, and Me* (2012) (Figure 1)—to collaborate with us, and she agreed!

The curatorial and research team for this project asked, “If graphic medicine is important enough to be featured in a new exhibition, should NLM also consider collecting works of this type?” At the time, NLM’s collection development staff took the opportunity to learn more about graphic medicine and, again, to consider collecting it.

## EXPLORATION OF THE GENRE

Forney collaborated with the NLM collection development staff in its exploration. Forney is active in the comics field; she is an educator and advocate for the power of graphic medicine. She identified forty-eight titles for NLM to review. Library staff also examined bibliographic databases, publisher and vendor title lists, and graphic medicine websites to identify additional selections for the collection.

Initially, collection activity focused on widely disseminated works, such as Roz Chast’s *Can’t We Talk about Something More Pleasant*? (2014), a renowned cartoonist’s memoir of caring for her aging parents. Staff sought works—some humorous, some harrowing, but all engaging—by patients, caregivers, and health professionals from diverse backgrounds and with unique viewpoints. The intention was not to collect all graphic medicine titles, but to assemble a representative collection that would likely have long-term appeal. To round out the collection, staff also acquired scholarly monographs about the genre.

While some graphic titles are available in print, many worthy titles exist only on the web. NLM has begun planning for the addition of graphic medicine to its web collecting and archiving program that utilizes the Archive-It web archiving service. It will join other NLM web collections, such as Global Health Events, Health and Medicine Blogs and Disorders of the Developing and Aging Brain: Autism and Alzheimer’s on the Web.

## FRAMING OF A COLLECTING PHILOSOPHY

The purpose of NLM, as outlined in the US Code, is “to assist the advancement of medical and related sciences and to aid the dissemination and exchange of scientific and other information important to the progress of medicine and to the public health” [2]. As new medical concepts, approaches, and disciplines proliferate, NLM assesses the worthiness of the associated literature within the framework of the *Collection Development Policy of the NLM*, fourth edition, which lists five criteria for selection. The materials must:

- Record progress in research in biomedicine and the related areas of the life sciences,
- Document the practice and teaching of medicine broadly defined,
- Demonstrate how health services are organized, delivered and financed,
- Chronicle the development and implementation of policy that affects research and the delivery of health services, and
- Illustrate the public perception of medical practice and public health. [3]

The team considering graphic medicine determined that this literature should be part of the NLM collection for the following reasons:

1. Graphic medicine records progress in research, especially from the perspective of the patient.
2. Increasingly, graphic medicine makes a dynamic contribution to the medical education of physicians, especially in medical humanities curricula. Through graphic novels, physicians explore the creation of graphic narratives and learn about how the health care system is experienced by patients.
3. Graphic medicine is well positioned to expose the development and implementation of policies that affect the delivery of health services. With their emphasis on the receivers of health service—specifically, the patients—graphic novels are exceptional, clear focused, and straightforward.
4. Similarly, graphic medicine presents a frank description of the public’s perception of medical practice.

If the objective is to collect works that capture a wide range of health-related behaviors, trends, and viewpoints and to chronicle experiences and perspectives not found in scientific, technical, and professional medical publications, then graphic medicine not only conforms to the objective, but also plays a unique role in the corpus of medical literature.

## FINAL NOTES

The graphic medicine initiative is one example of the ways in which different program areas of NLM help to inform, strengthen, and expand the collection. Thus far, NLM has acquired some forty titles that are part of the graphic medicine genre and continues to seek additions to this fledgling collection. NLM hopes that medical literature patrons around the world will enjoy and learn from the many unique and fascinating graphic medicine works by patients, family members, and health professionals. These publications not only are of interest to contemporary readers, but also are likely to fascinate future historians, medical sociologists, and medical anthropologists. For more information, visit the online exhibition *Graphic Medicine: Ill-Conceived and Well-Drawn!* and search LocatorPlus for the subject “graphic novels.” Happy reading!
